# Implemented occupational health surveillance limits the spread of SARS-CoV-2 Omicron at the workplace

**DOI:** 10.3389/fmed.2022.910176

**Published:** 2022-08-30

**Authors:** João Silveira Moledo Gesto, Adriana Cabanelas, Bruna Farjun, Monique Cristina dos Santos, Antonio A. Fidalgo-Neto, Sergio N. Kuriyama, Thiago Moreno L. Souza

**Affiliations:** ^1^SESI Innovation Center for Occupational Health, Rio de Janeiro, Brazil; ^2^Laboratório de Imunofarmacologia, Instituto Oswaldo Cruz, Fundação Oswaldo Cruz, Rio de Janeiro, Brazil; ^3^Center for Technological Development in Health (CDTS), Oswaldo Cruz Foundation (Fiocruz), Rio de Janeiro, Brazil; ^4^SENAI Innovation Institute for Green Chemistry, Rio de Janeiro, Brazil; ^5^National Institute of Science and Technology for Innovation on Diseases of Neglected Populations (INCT/IDN), Rio de Janeiro, Brazil

**Keywords:** COVID-19, SARS-CoV-2, occupational health, next generation sequencing – NGS, genomic surveillance

## Abstract

The global spread of the severe acute respiratory syndrome coronavirus 2 (SARS-CoV-2) has put an enormous pressure on human societies, at both health and economic levels. Early diagnosis of SARS-CoV-2, the causative agent of 2019 coronavirus disease (COVID-19), has proved an efficient method to rapidly isolate positive individuals and reduce transmission rates, thus alleviating its negative impact on society’s well-being and economic growth. In this work, through a coordinated and centralized effort to monitor SARS-CoV-2 circulation in companies from the State of Rio de Janeiro, Brazil, we have detected and linked an early rise of infection rates in January 2022 to the introduction of the Omicron variant of concern (VoC) (BA.1). Interestingly, when the Omicron genomic isolates were compared to correlates from public datasets, it was revealed that introduction events were multiple, with possible migration routes mapping to: Mali; Oman and United States; and Italy, Latin America, and United States. In addition, we have built a haplotype network with our genomic dataset and found no strong evidence of transmission chains, between and within companies. Considering Omicron’s particularly high transmissibility, and that most of our samples (>87%) arose from 3 out of 10 companies, these findings suggest that workers from such environments were exposed to SARS-CoV-2 outside their company boundaries. Thus, using a mixed strategy in which quick molecular diagnosis finds support in comprehensive genomic analysis, we have shown that a successfully implemented occupational health program should contribute to document emerging VoC and to limit the spread of SARS-CoV-2 at the workplace.

## Introduction

Over the last two and a half years, the surge and global spread of the severe acute respiratory syndrome coronavirus 2 (SARS-CoV-2) has been associated with approximately 6.3 million deaths worldwide (official numbers by June 2022)^1^ (1, 2). The burden of 2019 coronavirus disease (COVID-19), nonetheless, extends beyond death rates, with high hospitalization records in both primary and intensive care units, long-lasting sequelae, and an ever-growing impact on society well-being.

Economy-wise, COVID-19 has put an enormous pressure on industrial plants and local business, increasing work absence and affecting supply chains at national and international levels. In low- and middle-income countries, such as those in South America and Africa, the pandemic effect meets existing constraints and accentuates depression, urging solutions to better control and mitigate virus transmission.

Currently, the prompt diagnosis and isolation of SARS-CoV-2 positive individuals has been the standard goal of public health initiatives and the private sector, with a variable degree of success. In part, this is due to the nature of COVID-19 clinical evolution, and SARS-CoV-2 silent transmission by asymptomatic and pre-symptomatic individuals, whose identification is mandatory for lowering disease-associated risk. In the occupational environment, a systematic routine mass screening of workers, regardless of their symptoms, could fulfill this need and provide upfront crucial data for decision-makers to preclude transmission chains and preserve a safe and productive workplace (3).

The implementation of an effective SARS-CoV-2 surveillance system benefits from previous experience with influenza monitoring (4). Here, State laboratory facilities usually share patients’ samples with National Influenza Centers (NIC) to provide further intel on prevalent variants, and determine future strategies (i.e., vaccine updates) with the World Health Organization (WHO) Collaborating Centers. For SARS-CoV-2, however, the real-world scenario is more challenging, with the emergence of variant of concern (VoC) with distinct pathogenesis and the ability to evade immune responses. Gamma (P.1 and related lineages), Delta (B.1.617.2 and AY-related), and the recently documented Omicron (B.1.1.529 and BA-lineages) were all characterized by adaptive mutations (5–8) that could rapidly replace existing variants, and partially overcome immunity conferred by previous infections and/or vaccines. *Per se*, the BA.1 subvariant of Omicron alone harbors a set of 32 mutations at the immunogenic spike gene (9), when compared to the original Wuhan strain, and has disseminated among global populations at an unforeseen pace (10).

SNP typing (3) and genomic analysis (11) of SARS-CoV-2 isolates add extra layers to surveillance, consisting of powerful tools to track virus evolution and predict outbreaks of new VoC. Recently, this paradigm has been successfully tested under the occupational health perspective in Rio de Janeiro, not only replicating the Gamma-Delta replacement in the state, but also highlighting its potential contribution to genomic databases, phylogenetic inference of emerging VoCs, and healthcare risk management (11). However, despite the unequivocal importance of such findings, the impact of implemented occupation health and surveillance programs still needs to be measured for quality assessments and future improvements.

In this work, we report the early identification and percent positivity of SARS-CoV-2 Omicron variant among industry and service workers of the state of Rio de Janeiro, in samples collected from the end of December 2021 to mid-January 2022, and investigate whether this cohort harbored evolutionary evidence to support currently implemented occupational health programs. Using viral genome analysis, we detect multiple introductions of SARS-CoV-2 Omicron in Rio de Janeiro and demonstrate limited chains of transmission at the workplace, providing supporting evidence for effective healthcare risk management and further encouraging data-driven decision-making for healthcare compliance at the company site.

## Materials and methods

### Ethics and patients

The SESI Innovation Center for Occupational Health (FIRJAN, Rio de Janeiro, Brazil) employs a mass testing program for industry and service workers of the State of Rio de Janeiro (RJ), as part of an array of measures to quickly respond to and minimize the social and economic impact of the COVID-19 pandemic. From April 2020 till present day, this initiative has already responded to over 100K RT-qPCR COVID-19 tests, streamlining a workflow of sampling, logistics, molecular detection, and reporting the results back to the companies in less than 24 h. For this study, we selected 10 companies across the state, with variable organizational size, structure and workforce mobility, to track and investigate COVID-19 cases throughout a pandemic wave of a new SARS-CoV-2 variant. From December 2021 to January 2022, nasopharyngeal swabs from workers, syndromic and non-syndromic, were sampled in a timely fashion (every 1–7 days, with longer intervals for non-syndromic workers). Sampling was preferably carried out in the company by certified staff nurses, except for those organizations lacking a healthcare department or equivalent facility (i.e., companies with less than 50 workers). In those cases, external nurses were assigned to the service. The collected samples were then transported to the SESI Innovation Center, in which a centralized molecular testing facility was operating. SARS-CoV-2 positive samples were promptly recorded and notified to the company, to allow proper risk-reduction management (e.g., leave from work and social isolation). These records were also used by this study to monitor incidence levels over time, and some were randomly selected for next-generation sequencing (NGS) and more comprehensive genomic analysis. Regulatory approval for this work was obtained from the National Committee of Research Ethics (CONEP) and the Ethics Committee of Hospital Universitário Clementino Fraga Filho (Protocol 4317270).

### SARS-CoV-2 molecular detection

Nasopharyngeal swab samples were collected in DMEM cell culture medium, and submitted to RNA extraction using the Absolutely Total RNA Purification Kit (Agilent Technologies) and the Bravo Automated Liquid Handling Platform (Agilent Technologies), according to manufacturers’ instructions. Total RNA extracts were screened by RT-qPCR, following CDC standard protocols for SARS-CoV-2 detection. Amplification reactions were performed with TaqPath™ 1-Step RT-qPCR Master Mix (Applied Biosystems), and primers and probes for viral targets, N1 and N2, and the housekeeping gene RNase P (endogenous control) (IDT, Cat #10006713). Samples were considered positive when the three targets were amplified with Cycle threshold value (Ct) below 40.

### SARS-CoV-2 next generation sequencing

Syndrome coronavirus 2 positive samples with Ct ≤25 (N1 marker) were randomly selected for next generation sequencing and genome analysis. The Ct cut-off was adopted to set an abundant, high-quality, RNA standard for improving the isolation of genomic sequences and maximizing coverage. SARS-CoV-2 complete genomes were obtained following an amplicon-based massively parallel sequencing with the ATOPlex SARS-CoV-2 Full Length Genome Panel v2.0 (MGI Tech Co., Shenzhen, China). Briefly, total RNA were reverse-transcribed into cDNA and submitted to amplification reactions for targeted enrichment and dual-indexing. Double-stranded cDNA libraries were then pooled, circularized, and digested into single-stranded analogs. Finally, ssCirDNA were converted to DNA nanoballs by rolling circle amplification and pair-end sequenced (100 nt) on the MGISEQ-200 platform (aka. DNBSEQ-G50) (MGI Tech Co., Shenzhen, China).

### SARS-CoV-2 genome assembly and assignment of variants

FASTQ raw genomic data were demultiplexed and submitted to a customized Galaxy workflow for the analysis of pair-end amplicon data, along with auxiliary input datasets such as SARS-CoV-2 reference sequence (Wuhan-hu-1 isolate, GenBank MN908947.3) and a BED file containing primer coordinates of MGI’s ATOPlex panel v2.0. FASTQ were preprocessed using FASTP v.0.20.1 to remove adapters and reads shorter than 50 bp (-l 50). Reference mapping and genome assembly were carried out with BWA-MEM v. 0.7.17, set to default “auto” selection of the algorithm to build BWT index, and “Illumina analysis” mode. Output BAM files were subsequently filtered by quality (-q 20) and reformatted with SAMTools view v.1.13, so as to exclude (-F) unmapped reads (and its mate pairs) and those not consisting of primary alignments. Additionally, reads were edited at primers bindings sites with iVar trim v.1.3.1 (-m 1 -q 0 -s 4 -e), and realigned to the reference genome with LoFreq v.2.1.5, adding indel qualities based on Dindel algorithm. Variants were called with iVar variants v.1.3.1 (-q 30 -t 0.51 –pass_only), and output VCF files were used to call consensus with bcftools v.1.10. SARS-CoV-2 consensus genomes were aligned and assigned to global outbreak lineages with Pangolin v.3.1.17 (12), and to Nextstrain clades with NextClade v.1.5.1 (13). The latter also provided quality check reports for each consensus sequence, and generated an output json file for subsequent phylogeny assessment in Auspice v.0.8.0^2^ (14, 15).

### Phylogeny

Our original samples and sequences deposited in the GISAID^3^ (EPI_ISL_11627542 to EPI_ISL_11627636) were compared using IQ-TREE Phylogenomic/evolutionary tree construction from multiple sequences (Galaxy Version 2.1.2+galaxy2). Maximum-Likelihood phylogenetic analysis was performed with GTR + F + I + G4 nucleotide substitution model (16), and the branch support was assessed with 1,000 replicates. Mega-X software was used for the tree analysis.

### Genetic diversity and haplotype network analysis

Neutrality tests and haplotype data were generated with DnaSP v6.12.03. The haplotype network was inferred with TCS Networks (17), and drawn with PopART v1.7.

## Results

### Early evidence of a new SARS-CoV-2 variant in companies of Rio de Janeiro, Brazil

To better understand the dissemination of SARS-CoV-2 at the workplace, in the context of risk-reduction measurements adopted by occupational health programs, we tracked early evidence of pandemic waves of new variants in industry and service companies of Rio de Janeiro (RJ). By analyzing public health and internal surveillance databases, the latter implemented by the SESI Innovation Center for Occupational Health (Firjan) as part of a RT-qPCR mass-testing institutional program to identify, report and monitor COVID-19 among workers, we were able to successfully interpret the state epidemiological context and anticipate the ascent of new cases as an indicator of new variants being disseminated.

**FIGURE 1:**
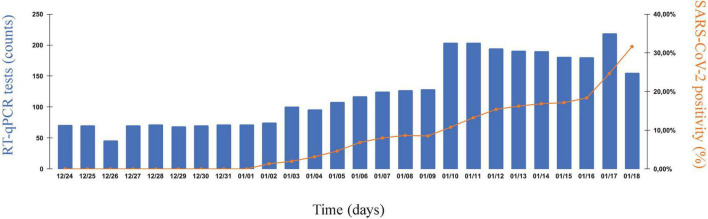
The new year uprise of SARS-CoV-2 cases in industrial and service companies of Rio de Janeiro. SARS-CoV-2 infection rates in nasopharyngeal swab samples collected from 25 December 2021 to 18 January 2022, as part of coordinated and continuous effort to monitor COVID-19 in companies of the state of Rio de Janeiro (RJ), Brazil. Samples were collected from syndromic and non-syndromic workers. Left vertical axis, number of SARS-CoV-2-specific RT-qPCR tests performed daily. Right vertical axis, SARS-CoV-2 percent positivity index (%). Horizontal axis, chronological time (as consecutive days of monitoring).

We analyzed a short interval of our internal surveillance data, spanning the end of December 2021 to mid-January 2022, and corresponding to COVID-19 cases recorded across 10 companies of RJ (Figure 1). Here, we could observe a shift from basal, virtually ‘0’, SARS-CoV-2 percent positivity to a dramatic uprise, with the onset around 2 January 2022. Additionally, the increment in percent positivity accompanies a higher demand for tests, mostly due to symptomatic workers. Overall, 3,553 tests were analyzed, 573 of which were positive.

### Characterization of SARS-CoV-2 variants related to the uprising cases

To investigate whether this wave of transmission was caused by a newly circulating variant of SARS-CoV-2, or the rebound of previously reported ones (e.g., Delta), we randomly selected 95 positive samples for further genomic analysis. This cohort had similar demographic and clinical characteristics to the total individuals tested in the same time period. Full-length SARS-CoV-2 genome sequences were isolated by targeted NGS, using a customized panel of amplicons (see section “Materials and methods”), and assigned to phylogenies for variant calling.

**FIGURE 2:**
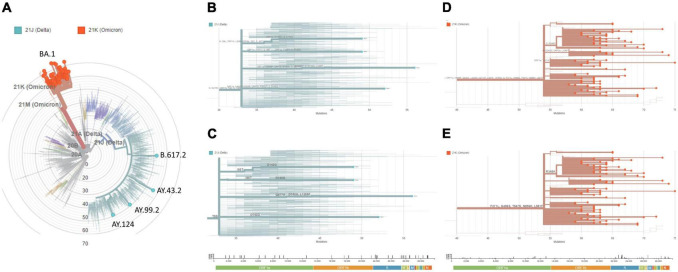
Genomic surveillance links uprising cases to Omicron VoC. From the cohort of SARS-CoV-2 positive samples, 95 were randomly chosen for targeted next-generation sequencing (ATOPLEX SARS-CoV-2 panel v2.0, MGI Tech Co.) and genomic analysis. **(A)** Maximum-Likelihood phylogeny of the isolated genome consensus sequences and publicly available datasets under NextClade v.1.5.1. **(B)** Diagram of mutations across the genome, or **(C)** restricted to the Spike gene, in cases associated with the Delta variant (clade 21J). **(D)** Diagram of mutations across the genome, or **(E)** restricted to the Spike gene, in cases associated with the Omicron variant (clade 21K). Genetic diversity (entropy panel) across the genome, whose components are color-coded, are represented below the diagrams. The genome consensus sequences isolated in this study were deposited at GISAID (https://www.gisaid.org; EPI_ISL_11627542 to EPI_ISL_11627636) and GenBank (https://www.ncbi.nlm.nih.gov/genbank; ON241656 to ON241750).

Our analyses revealed that only four samples were related to Delta (Nextstrain clade 21J) (Figure 2A). Pangolin classified those to lineages AY.43.2, AY.99.2, AY.124, and B.617.2, each with a characteristic set of mutations across the genome (Figure 2B), including some at the Spike gene (Figure 2C). The other 91 samples were related to the emerging Omicron variant (Nextstrain clade 21K), all of which were assigned to pangolin lineage BA.1 (aka. B.1.1.529.1) (Figure 2A). Here, a distinct set of mutations arose (Figure 2D), many of which were at the Spike (Figure 2E). Interestingly, these data corroborate recent reports suggesting that despite Omicron surging after Delta, and co-existing to some extent, these lineages fit into different clades and do not share a recent common ancestor at the evolutionary time scale (18).

**FIGURE 3:**
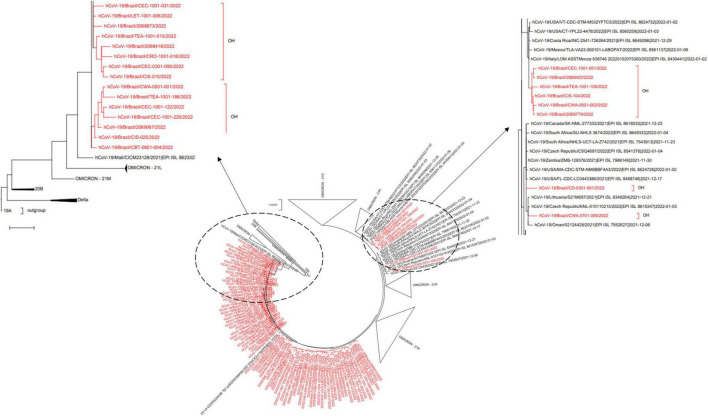
SARS-CoV-2 phylogeny reveals multiple introductions of Omicron VoC in Rio de Janeiro. The genome consensus sequences isolated in this study were compared to Omicron correlates deposited at GISAID (https://www.gisaid.org; Supplementary datasheet 1), using a Maximum-Likelihood phylogeny with GTR + F + I + G4 nucleotide substitution model, and *bootstrap* set to 1,000 replicates for tree branch support. The original Wuhan-Hu-1 genomic isolate was set as outgroup (clade 19A).

To further investigate the origins of Omicron dissemination in the state, using industry workers as sentinels, we compared our data with other genome sequences assigned to the same variant and publicly available at GISAID (see text footnote 3; Supplementary datasheet 1). Interestingly, the inferred phylogeny suggested multiple introductions (Figure 3), with putative migration events from: (a) Mali, (b) Oman and United States, and (c) Italy, Latin America, and United States.

### Implemented occupational health may limit SARS-CoV-2 spread among workers

**FIGURE 4:**
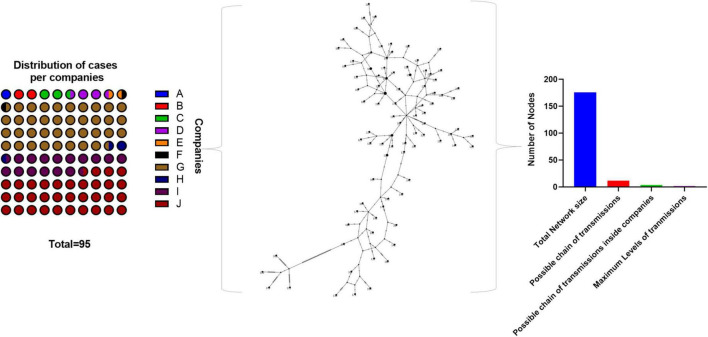
Implemented SARS-CoV-2 programs impairs transmission at the workplace. Isolated SARS-CoV-2 genome sequences were distributed across their respective companies of origin, and subsequently analyzed into a haplotype network. Interacting nodes were quantified according to different criteria, to reveal possible transmission chains, between and within companies. The haplotype network was inferred with the TCS method (i.e., statistical parsimony).

We have also analyzed our genomic data from a haplotype network perspective, in order to reveal signs of transmission chains occurring within companies (Figure 4). As one could notice, genomic data was not evenly distributed among the ten companies, named A to J, analyzed. Three of them – G, I, and J – were clearly overrepresented with more than 87% of the available genomes, suggesting that transmission evidence, if any, would probably be revealed here. In the haplotype network, a more comprehensive analysis suggested that possible transmission chains were rare, assigning only 12 out of 176 interacting nodes. And if only interactions within the same company were considered, transmission chains were even rarer, with 4 possible events occurring, suggesting that occupational health policies were effectively implemented.

## Discussion

To limit the spread of SARS-CoV-2 at the workplace, companies have relied on several measures to rapidly detect and isolate positive individuals. Random, non-selective, sensitive testing of all staff on a weekly basis, along with engagement campaigns for the adoption of effective non-pharmacological methods (i.e., masks, hand-sanitization, etc.), could represent a simple strategy for monitoring and controlling disease outbreaks. However, the real-world scenario is challenging, with companies organizing their space settings and workforce in variable levels of complexity, and providing self-limited resources for mass-testing and other extraordinary expenses. Solutions aimed at reducing the number of tests, and yet maintaining detection sensitivity, could circumvent the budget factor, thus broadening the implementation of mass testing programs. By learning and mapping the risk of infection across a company, for instance, testing efforts could be converged to fewer individuals. Here, the risk scale is intrinsic to each individual, and may derive from internal (company settings) and external factors (home settings, commuting to work alternatives, personal habits, and activities). One such solution, in which these factors are fed to an AI algorithm, has already been developed and successfully implemented by us to support the mass testing program in companies of the state of Rio de Janeiro, increasing the throughput and reducing costs. A straight-forward add-on was the pooling of asymptomatic samples, with individual re-testing restricted to positive pools (19, 20). Here, statistical modeling predicted a cost saving of 48% when prevalence rates reach around 7.5% and a four-samples pooling strategy is adopted. Indeed, in our mass-testing routine, this strategy accounts for a cost saving of about 51%, already considering subsequent re-testing of individual samples and false-negative rates (20).

Given the importance of monitoring and containing COVID-19 cases at the workplace, innovative strategies to better understand disease dynamics are crucial to fine-tune these processes. The advent of specific SARS-CoV-2 panels for NGS high-throughput data acquisition (21–24), coupled with powerful bioinformatics workflows, has provided an increasing amount of available genomic data and opened new possibilities for research and surveillance methods. Indeed, as a rapidly evolving pathogen, SARS-CoV-2 urges effective tracking of variants and continuous updates on their dissemination rates and clinical outcomes by public health institutions. Notwithstanding, the epidemiological scenario of a given population, either from a city or even from a whole state, may not reflect that of a specific company or industry sector, each with a particular organization structure. Thus, the implementation of genomic data analysis to current SARS-CoV-2 monitoring efforts could fill this gap and provide extremely valuable intel for companies, reflecting on more comprehensive quality metrics and better management of occupational health policies (11).

In this work, we have reported the spread of SARS-CoV-2 in 10 companies of the state of Rio de Janeiro, Brazil, from the end of December 2021 to mid-January 2022, revealing the identity of underlying genomic variants. We have shown that ongoing monitoring efforts could detect an uprising in COVID-19 percent positivity in early January 2022 (Figure 1), and that this phenomenon was mainly attributed to the recently documented Omicron variant (Figures 2, 3). These results are in line with publicly available genomic data of state samples collected during the same time period, reinforcing the overall prevalence of this VoC.^4^

As most of the COVID-19 cases arose from the companies “G,” “I,” and “J,” we wondered whether there could be internal transmission chains involved, triggered by their intrinsic organizational features and workforce transit. Interestingly, “G” is an Oil and Gas company, with intense mobility of the workforce between the office, offshore facilities and overseas subsidiaries; “I” is a multinational company manufacturing fluid sealing products, with one industrial plant in the state capital (three in Brazil), and moderate workforce mobility; and “J” is a not-for-profit organization, with a broad range of industry-related activities (e.g., education, research and innovation, administration, and consulting), and workforce mobility mostly restricted to the metropolitan area of RJ. As one could notice, these companies belong to different activity sectors, each with particular organizational features and workforce mobility requirements. It is clear, however, that companies like “G” inherit a higher internal transmission risk due to their increased workforce mobility, aggravated by further confinement in offshore facilities. However, when we examined our genome dataset arranged into a haplotype network, we had few interaction events suggesting very low internal transmission between and within these companies (Figure 4). Considering the high-risk level associated with some, this outcome was indicative that transmission chains were impaired by other factors.

One such factor could be the immunity offered by vaccination or previous exposure to SARS-CoV-2 (i.e., natural infection). Unfortunately, our data were not controlled for these variables due to ethical aspects, and we could not further investigate on interaction effects. Nonetheless, considering Omicron’s high transmissibility (9, 10), which partially overcomes cellular and humoral immune protection, it is unlikely that this factor alone limited transmission. The social isolation of positive individuals, as well as the adherence to non-pharmacological prevention methods at the workplace (e.g., masks, hand sanitization, measures to promote air renovation, and decrease human density), even though not measured empirically, were possibly contributing to a greater extent. With little evidence of transmission chains, it is presumable that the COVID-19 cases in our cohort arose from outside the workplace, and containment measures adopted by occupational health programs were effective to avoid transmission from the inside.

Overall, our study demonstrates that COVID-19 monitoring programs implemented by industry and service companies could benefit from genomic data to build a clearer picture of the pandemic within their boundaries. Data of this nature are often unique, reflecting multilevel interactions between populations of different cities, states, and countries due to mobility requirements. Thus, workers may often represent good sentinels for tracking new variants, and a good starting point for containment policies. In our study cohort, we could successfully detect the early Omicron circulation in the state of Rio de Janeiro, employing a similar strategy to that used to report the Gamma/Delta replacement during mid-2021 (11). In addition to contributing to the surveillance of variants, genomic data can also reveal potential transmission chains under course in some company settings, providing quality control metrics for tightening or loosening occupational health measures. As a COVID-19 monitoring center, we witnessed surprisingly low transmission indexes in our haplotype network analysis of state company samples, suggesting that internal policies met appropriate standards for a safe workplace, and paving the way for this kind analysis as part of the pathogen management toolkit in occupational health. One could speculate that programs embracing such strategy shall be able to fine-tune measures that directly impact productivity, preserving workers’ well-being while avoiding excessive policies. In the real-world, however, the feasibility of genomic data analysis and development of related tools for the market still face some challenges, including the overall cost, the time consumed by NGS protocols and availability of efficient data processing infra-structure. In addition, its implementation presumes well-established ethical and regulatory documentation, including informed consent of workers’ genomic data sampling, analysis, storage, with privacy and security compliance assured. Thus, future initiatives aimed at these targets shall contribute to level the access and make the implementation of these tools more realistic worldwide.

## Data availability statement

The original contributions presented in this study are publicly available. This data can be found here: Genbank repository, accessions ON241656–ON241750.

## Ethics statement

The studies involving human participants were reviewed and approved by the National Committee of Research Ethics (CONEP) and the Ethics Committee of Hospital Universitário Clementino Fraga Filho (Protocol 4317270). The patients/participants provided their written informed consent to participate in this study.

## Author contributions

SK, AF-N, and TS conceptualized the study. JG, AC, BF, MS, SK, and TS performed the investigation, data curation, and analysis. SK and TS managed the project supervision, validation, and funding. All authors drafted the manuscript, reviewed, and approved its final version.

## Footnotes

1
https://arcg.is/0fHmTX

2
https://auspice.us/

3
https://www.gisaid.org

4
https://www.genomahcov.fiocruz.br/dashboard-pt/
